# Tissue-Specific Roles of NKT Cells in Tumor Immunity

**DOI:** 10.3389/fimmu.2018.01838

**Published:** 2018-08-15

**Authors:** Masaki Terabe, Jay A. Berzofsky

**Affiliations:** Vaccine Branch, Center for Cancer Research, National Cancer Institute, National Institutes of Health, Bethesda, MD, United States

**Keywords:** NKT cell, type II NKT cell, iNKT cells, tumor immunology, immune regulation, immune network, tissue-specific immune response, tissue-resident cells

## Abstract

NKT cells are an unusual population of T cells recognizing lipids presented by CD1d, a non-classical class-I-like molecule, rather than peptides presented by conventional MHC molecules. Type I NKT cells use a semi-invariant T cell receptor and almost all recognize a common prototype lipid, α-galactosylceramide (α-GalCer). Type II NKT cells are any lipid-specific CD1d-restricted T cells that use other receptors and generally don’t recognize α-GalCer. They play important regulatory roles in immunity, including tumor immunity. In contrast to type I NKT cells that most have found to promote antitumor immunity, type II NKT cells suppress tumor immunity and the two subsets cross-regulate each other, forming an immunoregulatory axis. They also can promote other regulatory cells including regulatory T cells (Tregs) and myeloid-derived suppressor cells (MDSCs), and can induce MDSCs to secrete TGF-β, one of the most immunosuppressive cytokines known. In some tumors, both Tregs and type II NKT cells can suppress immunosurveillance, and the balance between these is determined by a type I NKT cell. We have also seen that regulation of tumor immunity can depend on the tissue microenvironment, so the same tumor in the same animal in different tissues may be regulated by different cells, such as type II NKT cells in the lung vs Tregs in the skin. Also, the effector T cells that protect those sites when Tregs are removed do not always act between tissues even in the same animal. Thus, metastases may require different immunotherapy from primary tumors. Newly improved sulfatide-CD1d tetramers are starting to allow better characterization of the elusive type II NKT cells to better understand their function and control it to overcome immunosuppression.

## Introduction

NKT cells are a small population of true T cells which are distinct from conventional T cells in that their receptor recognizes lipids rather than peptides and is restricted by a non-classical class I-like (class Ib) molecule CD1d (1–8). The term NKT derives from early work in which NK1.1 was used as a marker and evidence that they were early responders like innate NK cells (so “natural killer T cells”), but as many of them are NK1.1 negative, the definition has changed to require just recognition of lipid or glycolipid antigens presented by CD1d (2). This property gives NKT cells a special place in the immune system. As part of the adaptive immune system, they give the adaptive T cell immune system a way to recognize lipids, to which it is otherwise blind, as conventional MHC molecules are limited in general to presentation of peptide fragments of proteins. They also function as part of the innate immune system in that they have pre-formed mRNA for cytokines, allowing rapid cytokine production, and are among the first responders in many infectious and inflammatory processes, similar to innate cells (9, 10). Their early cytokine production can potentially set the tone for other adaptive immune responses to follow.

A key discovery was the use of a semi-invariant T cell receptor (TCR) by NKT cells, characterized by semi-invariant TCRα using Vα14Jα18 gene segments together with Vβ8, 7, or 2 in mice, and Vα24Jα18 with Vβ11 in humans (Table 1). This was first detected in T cell hybridomas (11, 12) and subsequently in a subset of primary T cells (12–17). Thus, when the Cardell et al., first identified CD1d-restricted T cells that lacked this semi-invariant TCR as residual CD4^+^ T cells present in MHC class II-deficient mice (18, 19), these were called non-classical or type II NKT cells and those with the semi-invariant TCR became known as classical or type I NKT cells, or iNKT cells for invariant NKT cells (2).

**Table 1:** NKT cell subsets.

	Type I or semi-invariant						Type II
TCRα chain	Vα14Jα18 (mice)						Diverse
	Vα24Jα18 (humans)						
TCRβ chain	Vβ2, 7, 8 (mice)						Diverse
	Vβ11 (humans)						
Representative agonists	α-GalCer, β-ManCer						Sulfatide
Transcriptional factor	PLZF						PLZF?

Functional subtypes	NKT1	NKT2	NKT17	NKT10	NKT_FH_	NKTreg	Not known
Transcriptional factor	T-bet	(PLZF^hi^)	RORγ-t	E4BP4	Bcl-6	Foxp3	Not known
Cytokines	IFN-γ	IL-13	IL-17	IL-10	IL-21	?	IL-4, IL-13, IFN-γ

Another key discovery was that virtually all type I NKT cells responded to a common prototype lipid, α-galactosylceramide (α-GalCer), originally isolated from a marine sponge but probably derived from microbes symbiotic with the sponge (20) (Table 1). This greatly facilitated studying the function of type I NKT cells and also led to the development of a marker for these, α-GalCer-CD1d tetramers by the Kronenberg, Bendelac, and Cerundolo labs (21–23). The tetramers filled a key role since NK1.1 was no longer a meaningful marker to characterize these T cells. However, in contrast, type II NKT cells were defined by what they were not, i.e., they were CD1d-restricted T cells that did not use the semi-invariant TCR and did not recognize α-GalCer. The activity of type II NKT cells was primarily studied by comparing wild-type mice that had both types with Jα18^−/−^ mice that lacked only type I NKT cells and CD1d^−/−^ mice that lacked both (24). Subsequently, the Kumar lab discovered recognition of sulfatide from myelin sheaths in the central nervous system by type II NKT cells and developed sulfatide-loaded-CD1d tetramers to detect this subset of type II NKT cells (25–27). Presumably, these do not detect all type II NKT cells because the repertoire is more diverse, but they have become the best studied subset. Although production and use of sulfatide tetramers has not been as straightforward as for α-GalCer-loaded-CD1d tetramers, they have been useful to characterize this subset of type II NKT cells, and our lab has developed more stable sulfatide-CD1d tetramers (28).

Besides dividing NKT cells into subsets based on their TCRα, whether or not they use Vα14Jα18 TCRα, NKT cells, especially type I NKT cells, can be divided based on their surface marker expression, cytokine production, and transcription factor expression. Historically, much effort has been made to use surface markers to identify functional subsets of type I NKT cells (29–34). However, there is some limitation using surface markers since some markers, such as NK1.1, are expressed in limited strains of mice. Therefore, Hogquist’s lab proposed to use transcription factors, T-bet, PLZF, and RORγ-t, to distinguish functional subsets of type I NKT cells, especially in the thymus (35). They showed that analogous to CD4^+^ T cells subsets, type I NKT cells can be divided into NKT1, NKT2, and NKT17 functional subsets that correspond to Th1, Th2, and Th17, respectively (35, 36). Now more subsets, IL-10 producing E4BP4^+^NKT10 (37, 38), T_FH_ like NKT_FH_ (39, 40) and regulatory T cell (Treg)-like Foxp3^+^NKTreg (41) have been reported (Table 1). Although their roles in tumor immunity are not clear yet, it is of interest to explore which subsets are responsible for the functions of NKT cells in tumor immunity discussed below.

## Type I NKT Cells in Tumor Immunity

Type I NKT cells may have evolved in part to recognize certain bacterial pathogens, especially ones that lack LPS to alert the immune system, as lipids from *Sphingomonas, Erlichia*, and *Borrelia*, presented by CD1d, have been found to be recognized by the semi-invariant TCR of type I NKT cells (42–45). They can also be activated by LPS-expressing bacteria through non-antigen-specific signals like IL-12 (43, 46) or a combination of IL-12 and IL-18 (47). They can also ameliorate autoimmune diseases such as diabetes (48–50) or experimental autoimmune encephalitis (51), or exacerbate them, such as hepatitis (27, 52).

The focus of this review is NKT cells in cancer, where they also play a critical role. While the role of type I NKT cells in protection against autoimmunity has been mainly through production of Th2 cytokines like IL-4 and IL-13, their protection against cancer has been found to be largely dependent on production of Th1 cytokines, especially interferon-γ (IFN-γ), even though NKT cells have lytic activity and could potentially directly lyse tumors that express CD1d (53, 54). This has been observed both in studies involving treatment with α-GalCer and its analogs and in studies of spontaneous immunosurveillance without an applied treatment. Indeed, α-GalCer was observed to have potent antitumor activity (55–57) even before it was discovered to be a potent agonist for type I NKT cells (20). Dendritic cells (DCs) pulsed with α-GalCer were also found to be therapeutic against established liver metastases of the B16 melanoma and had the advantage that they were less able to induce anergy of NKT cells (58, 59). Analogs of α-GalCer that were skewed more toward IFN-γ induction, such as C-glycoside, were even more potent (60). Furthermore, the protection afforded by α-GalCer was dependent on the sequential induction of NK cells by the NKT cells, both of which could produce IFNγ and provide lytic antitumor activity (61, 62). Even rejection of tumors induced by low doses of IL-12 was found to be dependent on type I NKT cells by the failure to protect Jα18^−/−^ mice (17, 63–65).

NKT cells have been implicated in protective tumor immunosurveillance in the absence of an applied variable, by comparing tumor formation in wild-type vs Jα18^−/−^ mice. For example, immunosurveillance against methylcholanthrene-induced sarcomas in mice was found to be deficient in Jα18^−/−^ mice (66). This study also found that NKT cells were critical for protection in models of immunosurveillance dependent on exogenous production of IL-12, whereas others were more dependent on NK cells. This finding is consistent with the discovery that another mechanism by which NKT cells can promote tumor immunity is by activating DCs to make IL-12, which is a potent inducer of IFN-γ (67) and to be more effective at inducing CD4^+^ and CD8^+^ T cells (68). The Jα18^−/−^ mice could be reconstituted with purified wild-type liver NKT cells to protect the knockout mice, proving that the protection was dependent on NKT cells (69). Moreover, in that model, the CD4/8 double-negative subset of liver NKT cells was most protective, whereas those from thymus and spleen were less so, implying different activities of different subsets of type I NKT cells from different tissues (70). Similar findings were made for reconstitution of Jα18^−/−^ mice with NKT cells to protect against lung metastases of a BALB/c sarcoma (71). In this case, interestingly, CD4^+^CD25^+^ Tregs were induced that seemed to inhibit protection primarily by decreasing the number of NKT cells in the lungs.

NKT cells express perforin and granzymes in lytic granules, and human NKT cells have been found to use these to lyse a variety of tumor cells *in vitro* (72). In addition, a major mechanism of killing by NKT cells *in vivo* was found to be through Fas–FasL interaction (73). Nevertheless, other studies have found that a major protective mechanism of NKT cells against cancer involves production of IFN-γ and induction of other effector cells downstream, especially NK cells and CD8^+^ T cells. For example, protection against the methylcholanthrene-induced tumors by adoptive transfer of wild-type NKT cells into Jα18^−/−^ mice required their ability to make IFN-γ but not perforin, and on induction of NK cells that did need to be capable of making perforin (69). Moreover, sequential production of IFN-γ first by NKT cells and then by NK cells was necessary (61, 62). NK cell induction by NKT cells is rapid (74) and depends on IL-2, IFN-γ, and in some situations IL-21 (62, 75).

Thus, the major mechanisms by which type I NKT cells protect involve several pathways, production of IFN-γ, activation of DCs to make IL-12 and also be more effective antigen-presenting cells, and then downstream activation of NK cells and CD8^+^ T cells that also make IFN-γ and mediate tumor lysis. This appears to apply to most of the α-GalCer analogs that have been studied.

An exception comes from studies in our lab which identified an unusual analog, β-mannosylceramide (β-ManCer) that differs in both the sugar (mannose instead of galactose) and the linkage (β instead of α), which appears to protect against lung metastases in mice by a different mechanism and is considered the first example of a new class of NKT cell agonists that work by a distinct mechanism (76, 77). We found that β-ManCer was a poor inducer of cytokines *in vivo* and *in vitro*, and protected even in IFN-γ^−/−^ mice. Rather, the protection was dependent on TNF-α and nitric oxide synthase (NOS) (76). The mechanism by which TNF-α and NOS interact to protect is under study. We also found that, like certain phenyl-glycolipids (78) that can be more efficacious against cancer than α-GalCer (79), β-ManCer induces little or no long-term anergy of NKT cells, in contrast to α-GalCer that induces anergy, so that the NKT cells cannot be re-activated by re-stimulation with α-GalCer or its analogs even 2 months later (77). This is important for repeated dosing if this is to be translated to the clinic. Also, because the mechanism is different, β-ManCer synergizes with α-GalCer to protect when both are used at sub-therapeutic doses (76).

In human cancer patients, defects have been observed in type I NKT cells. Numbers of these were reduced in cancer patients compared with healthy controls in a number of solid tumors (80). Also, production of IFN-γ by NKT cells was significantly decreased in multiple myeloma patients (81). High numbers of infiltrating type I NKT cells was a predictor of overall survival in colorectal cancer (82) and low circulating levels of type I NKT cells was a predictor of poor survival in head and neck squamous cell carcinoma (83). Attempts to use α-GalCer therapeutically in advanced human cancer have not been as successful as predicted from murine studies. Initial studies of α-GalCer itself (80) or autologous DCs pulsed with α-GalCer (84–86) could increase NKT cell numbers and/or cytokine levels, and appeared safe. Expansion of patient’s type I NKT cells *ex vivo* and reinfusion also was safe and increased numbers (87). However, none of these treatments resulted in any complete or partial remissions of the cancer. More recent attempts at treatment with α-GalCer-pulsed DCs have achieved prolongation of median survival in lung cancer and some partial responses in head and neck cancer (88, 89). Studies are underway to use induced pluripotent stem cells to generate large numbers of autologous NKT cells for therapy (89).

## Type II NKT Cells in Tumor Immunity

In view of all the evidence above in both mice and humans that NKT cells play primarily a protective role in cancer, it came as a surprise when we discovered that NKT cells could also suppress tumor immunosurveillance (90). A BALB/c fibrosarcoma (15-12RM) that expressed the HIV envelope protein grew, regressed, and then recurred in almost all the mice, but failed to recur in CD1d^−/−^ mice lacking NKT cells. We traced this to production of IL-13 by the NKT cells that induced myeloid cells (a CD11b^+^ Gr1 intermediate population, probably a form of myeloid-derived suppressor cell or MDSC) to make TGF-β, and it was the TGF-β that suppressed the CD8^+^ T cell-mediated protection (90, 91). Blockade of either IL-13 or TGF-β or elimination of either the NKT cell or the myeloid cell could interrupt this immunosuppressive circuit and unmask immunosurveillance, preventing the tumor recurrence. The same was true in a CT26 colon cancer lung metastasis model. A puzzle in this pathway was why IL-13 but not IL-4 was necessary, when the protection depended on both the IL-4Rα and STAT6, which are downstream of both IL-4 and IL-13 (90). The solution to this puzzle was found when it was discovered that the signal from IL-4Rα and STAT6 synergized with a signal from TNF-α to upregulate the IL-13Rα2, a second receptor for IL-13 that does not respond to IL-4, and this latter receptor, when triggered by IL-13, induced the myeloid cell to make TGF-β (92). Another concurrent study also found that immunosurveillance was revealed in STAT6-deficient mice, consistent with our findings (93, 94). Nevertheless, in another mouse tumor model, IL-4Rα, STAT6, and TGF-β were not required for immunosuppression by NKT cells, implying that other suppressive mechanisms also exist (95).

The paradox that NKT cells both promoted and suppressed tumor immunity was resolved when it was found that type II NKT cells, still present in Jα18^−/−^ mice but absent in CD1d^−/−^ mice, were sufficient to suppress (24). This was true in several tumor models studied in different labs, in which Tregs did not play a critical role. A similar finding that type I NKT cells promoted tumor immunity and type II NKT cells suppressed it was observed in a B-cell lymphoma model (96).

As noted above, the type II NKT cells have been difficult to study due to lack of a good surface marker, with the sulfatide tetramers being difficult to make and use. We have now made stable sulfatide-CD1d tetramers and have been characterizing the type II NKT cells which seem to be enriched in both lung and liver, two tissues that are major targets for tumor metastases (28) (also Kato, Pasquet et al., submitted). This is especially interesting as we have seen the role of type II NKT cells in lung metastasis models in syngeneic mice (24, 91). The question of tissue specificity and tissue microenvironment will be discussed further below.

We have also focused on the suppressive role of TGF-β that is the downstream immunosuppressive cytokine in this pathway. Because it is also involved in Treg induction and function and is also made by tumors, we cannot attribute all the effects of TGF-β blockade to the NKT cell immunoregulatory pathway, but some of it may be due to that pathway. Conversely, blockade of TGF-β could block multiple regulatory pathways simultaneously. It is also possible that TGF-β induced by NKT cells from myeloid cells mediates a negative feedback loop on the NKT cells themselves, as it has been shown that TGF-β downregulates antigen presentation by CD1d by antigen-presenting cells without reducing the level of surface CD1d (97). Blockade of TGF-β unmasked tumor immunosurveillance in both the 15-12RM subcutaneous fibrosarcoma model and the CT26 lung metastasis model in the absence of any other treatment (91). In the TC1 model of an HPV E6/E7 transformed tumor, and in a CT26 subcutaneous tumor model, TGF-β blockade did not protect by itself, but synergized with a vaccine to reduce tumor growth more completely than the vaccine alone (98, 99). Furthermore, blockade of only TGF-β1 and β2 isoforms, without blockade of TGF-β3, was sufficient to mediate both effects (100). This may reduce the number or types of side effects. Also, importantly, as they work by different pathways, we found that anti-TGF-β enhanced the efficacy of anti-PD1 and *vice versa* for improving vaccine efficacy. Thus, the triple combination of vaccine, anti-PD1, and anti-TGF-β gave better protection than any of the possible pairwise combinations (100). This combinatorial efficacy is also supported by recent findings that one reason for failure of anti-PD1 therapy is TGF-β that inhibits T cell entry into the tumor in urothelial and colon cancers (101, 102). A phase I clinical trial of a human anti-TGF-β antibody in advanced metastatic melanoma patients also showed some preliminary evidence of beneficial activity, including an 89% partial response and several cases of mixed response or stable disease, with surprisingly few side effects (103, 104). All these results support the use of anti-TGF-β as a novel checkpoint inhibitor alone or in combination with vaccines and anti-PD1.

## Cross-Regulation Between Type I and Type II NKT Cells

If type I and type II NKT cells generally play opposite roles in cancer immunosurveillance, and type II NKT cells are often immunosuppressive, is it possible that type II NKT cells also suppress type I NKT cells and *vice versa*? We explored this question in mouse tumor models by taking advantage of the ability to selectively stimulate type I NKT cells with α-GalCer and type II NKT cells with sulfatide (105). In a CT26 colon cancer lung metastasis model, treatment of mice with α-GalCer protected against lung tumor nodules almost completely, whereas treatment with sulfatide actually increased the number of lung nodules relative to the vehicle control, consistent with promotion of tumor immunosurveillance by type I NKT cells and suppression of endogenous surveillance by type II NKT cells (105). By contrast, lack of type I NKT cells in Jα18^−/−^ mice led to increased tumor nodules, consistent with removal of a check on type II NKT suppression when type I NKT cells were absent, whereas CD1d^−/−^ mice that lack both were protected. Moreover, sulfatide stimulation of type II NKT cells suppressed proliferation and cytokine secretion of type I NKT cells in BALB/c spleen cells induced by α-GalCer. This was not due to competition for binding to CD1d, because APCs could be pulsed separately with α-GalCer and sulfatide and then mixed to achieve the same result. Also, *in vivo* stimulation of both increased the ratio of IL-13/IFN-γ compared with stimulation with α-GalCer alone. The key finding was that in two mouse tumor models, the subcutaneous 15-12RM fibrosarcoma model and the CT26 lung metastasis model, when animals were treated with both α-GalCer and sulfatide, the protective effect of α-GalCer was abrogated or reduced, implying that type II NKT cell stimulation inhibits protection by type I NKT cells (105). In a concurrent study, Vipin Kumar’s lab showed that sulfatide-stimulated type II NKT cells inhibited ConA-mediated hepatitis that was dependent on type I NKT cells (27).

These studies indicate the existence of an immunoregulatory axis between type I and type II NKT cells, in which they not only have polar opposite functions, but counteract each other (7, 54, 105–109). This axis is reminiscent, therefore, of the original Th1–Th2 immunoregulatory axis described by Mosmann and Coffman (110, 111) that had such a profound effect on immunology, because the cross-regulation set up a metastable state in which whichever polarized cells got a head start would suppress the other pole, and thus shift the balance to one pole or the other. Because NKT cells serve as first responders on the scene in many types of immune response, the balance along this type I–type II NKT cell immunoregulatory axis could similarly set the tone for subsequent other adaptive immune responses.

## Networking of NKT Cells with Other Regulatory Cells

### NKT Cells and Myeloid Cells

As a part of the large immune network, NKT cells also interact with other regulatory cells such as Tregs and MDSCs. In addition to the interaction between type II NKT cells and myeloid cells suppressing tumor immunity, type I NKT cells have been reported to interact with MDSCs. MDSCs are converted from expanded immature myeloid cells generated from normal progenitors of myeloid cells and neutrophils (112). They can be further differentiated into myeloid lineage cells such as DCs and macrophages. MDSCs can be converted into immunostimulatory APCs when the MDSCs present α-GalCer and tumor antigens (113). The interaction of α-GalCer-presenting MDSCs with type I NKT cells increased expression of CD11b, CD11c, CD40, MHC II, and CD86 on MDSCs, which are markers of stimulatory APCs. Inoculation of MDSCs loaded with α-GalCer and tumor antigen induced protective tumor immunity dependent on CD8^+^ T cells, NK cells, and type I NKT cells, but not CD4^+^ T cells and host DCs. Consistent with changes in the surface marker expression, the treatment did not suppress CD8^+^ T cells and did not induce Tregs. Although the detailed mechanism of conversion of MDSCs into stimulatory APCs is not clear, the effect of type I NKT cells on interacting MDSCs to induce maturation of the cells to become more stimulating myeloid cells is similar to what has been reported of interaction of type I NKT cells with α-GalCer-presenting immature DCs, which are induced to mature through CD40–CD40L interaction (114). NKT cells not only manipulate MDSCs but also allow CD8^+^ T cells to acquire resistance against MDSCs during *ex vivo* expansion (115). On the other hand, inoculation of cell free α-GalCer can induce accumulation of MDSCs through induction of IL-33 production by Kupffer cells (116). Type I NKT cells also modulate suppressive IL-10-secreting neutrophils induced by serum amyloid-A (SAA-1), an acute phase reactant (117). Signaling by SAA-1 also conversely facilitates the modulation of the neutrophils by type I NKT cells. This interaction downmodulates IL-10 production by neutrophils through a mechanism dependent on CD1d and CD40, which induces IL-12 production. Type I NKT cells can also control myeloid cells by directly lysing them, as they express CD1d. In a primary neuroblastoma without MYC-N amplification, CD1d-expressing CD68^+^ tumor-associated macrophages (TAMs) stimulate tumor growth through production of IL-6, and a high TAM gene signature is associated with poor prognosis (118). In a xenograft human neuroblastoma model in NOD/SCID mice, adoptively transferred type I NKT cells selectively killed TAMs by recognizing CD1d to indirectly control tumor growth (118).

By contrast, there are some studies suggesting myeloid cells suppress type I NKT cells in some situations. High neutrophil concentration was reported to suppress type I NKT cell activation and expression of T-bet both *in vivo* in CD18^−/−^ spontaneous neutrophilic mice and *in vitro* by co-incubating type I NKT cells with neutrophils (119).

### Treg and NKT Cells

In contrast to the relationship between type I NKT cells and myeloid cells, in which most studies demonstrated that type I NKT cells modulate suppressive tumor-associated myeloid cells, type I NKT cells facilitate and collaborate with Tregs to induce immune suppression in multiple settings including allergic asthma, type I diabetes, tolerance induction, and bone marrow transplantation (109, 120–125). Here, we focus the discussion on studies in the context of cancer.

Polyps are pathological sites of inflammation in intestine which subsequently often develop into cancers. Recently, Wang et al. reported that type I NKT cells drive Treg maintenance and/or activation in both polyps and lamina propria of APC^min/+^ mice, which has genetic disposition to develop spontaneous polyps (126). When APC^min−/+^ mice are made deficient for type I NKT cells, there is a significant reduction in the number of polyps. Type I NKT cell-sufficient APC^min/+^ mice have higher number of Foxp3^+^ Tregs with more activated phenotype in polyps compared with type I NKT cell-deficient APC^min/+^ mice. The existence of type I NKT cells also altered a myeloid cell population from iNOS-expressing M1 dominant macrophage phenotype in type I NKT cell-deficient animals to CD206-expressing M2 macrophage phenotype and Ly6G^hi^Ly6c^int^ MDSCs. Recognition of commensal bacterial antigens are critical to mature and maintain type I NKT cells in mucosal tissues (119). Cell-to-cell contact of type I NKT cells, weakly stimulated with bacterial antigens or cytokines, with Tregs induces IL-10 production and upregulation of Foxp3 in Tregs to make Tregs more suppressive in humans (127). Thus, it is possible that weakly stimulated type I NKT cells residing in the intestine tend to induce activated suppressive Tregs in the tissue, and this may serve as a mechanism of gut homeostasis.

As described above, type I NKT cells support Tregs both in mice and humans, while paradoxically Tregs suppress type I NKT cell functions. Human Tregs suppress proliferation and cytokine production of type I NKT cells when type I NKT cells are stimulated with relatively weak antigens such as OCH, an α-GalCer analog, or bacterial-derived diacylglycerol *in vitro* (127). The suppression is dependent on both cell-to-cell contact and IL-10. Presumably, IL-10 production induced by cell-to-cell contact between type I NKT cells and Tregs mediates the suppression of type I NKT cells by Tregs. *In vivo*, in the context of cancer, Tregs can suppress type I NKT cells and type I NKT cell-mediated tumor immunity in a model with a methylcholanthrene-induced tumor cell line (71). In this model, vaccination with tumor antigens induced tumor antigen-specific Tregs, reduction of the number of type I NKT cells and accelerated tumor growth. The suppression of type I NKT cells by Tregs affects NKT cell-targeted immunotherapy of cancer. Blockade of Tregs by anti-CD25 mAb treatment enhances the efficacy of DCs pulsed with α-GalCer and tumor-derived antigens to induce effector CD8^+^ T cells in a B16.OVA model (128). Similarly, transient depletion of Tregs in DEpletion of REGulatory cells (DEREG) mice, which express a diphtheria toxin receptor under a control of Foxp3 promoter/enhancer regions, by diphtheria toxin injection enhances the therapeutic effect of an α-GalCer-loaded tumor cell vaccine in a B16F10 melanoma model (129). In contrast to type I NKT cells, interaction between type II NKT cells and Tregs is not yet understood.

### Balance Between Type II NKT Cells and Tregs Determined by Type I NKT Cells

As we discussed above, there is cross-talk between type I and type II NKT cells forming an immunoregulatory axis. Also, there is a clear interaction between type I NKT cells and Tregs. What is the relationship between those two interactions?

In a subcutaneous CT26 colon carcinoma model, it has been shown that blockade of Tregs by anti-CD25 reveals tumor immunosurveillance that rejects transplanted tumors. The effect of anti-CD25 was similar in CD1d^−/−^ mice that do not have either type of NKT cells. However, it was not the case in Jα18^−/−^ mice that lack type I NKT cells but retain type II NKT cells (130), indicating that type II NKT cells are a second cell responsible for the suppression of tumor immunity in Jα18^−/−^ mice. The inability of anti-CD25 treatment to unmask immunosurveillance in Jα18^−/−^ mice could be reversed by two different approaches. One is blockade of antigen presentation by CD1d by treating mice with anti-CD1d mAb (130). This treatment blocks activation of type II NKT cells in Jα18^−/−^ mice *in vivo*, and no other cells since these mice lack type I NKT cells. The second is adoptive transfer of type I NKT cells into Jα18^−/−^ mice, allowing type I NKT cells to counter-regulate the type II NKT cells (105, 130). Both approaches made anti-CD25 treatment effective to induce tumor rejection in Jα18^−/−^ mice. In NKT cell-sufficient mice, counter-regulation between the two types of NKT cells cancels out their functions to a large extent, allowing Tregs to be the dominant regulator of tumor immunity. It is the status of type I NKT cells that determines dominance of Treg-mediated suppression. Indeed, tipping the balance between type I and type II NKT cells by skewing toward type II NKT cell dominance without removing type I NKT cells, by specifically stimulating type II NKT cells with sulfatide *in vivo*, also made anti-CD25 ineffective to remove immunosuppression of tumor immunity (130). This is clinically relevant because, as noted above, the type I NKT cell number in peripheral blood has been reported to be suppressed in cancer patients (80, 83, 131) compared with healthy donors, and their IFN-γ-producing function is frequently diminished in patients (81, 132–135), making the balance in cancer patients more like that in Jα18^−/−^ mice. In addition, it is believed that type II NKT cells are more prevalent in humans compared with mice. Therefore, in cancer patients, it is quite possible that imbalance of the type I–type II NKT cell axis may provide an explanation why Treg-targeted therapy development has had very limited success despite much effort invested to develop this type of immunotherapy (136).

## Tissue Specificity of Immune Regulation and Effector Function in Cancer

Tissue-resident immune cells such as innate lymphocytes and resident memory T (T_RM_) cells, mainly CD8^+^ T cells, have recently been implicated in tissue homeostasis and tissue-specific immunity against pathogens and cancer. Similar to the situation with innate lymphocytes, as NKT cells have one foot in the innate immune system, distribution of NKT cell functional subsets is distinct among different tissues (31, 32, 38, 119, 137, 138). Thus, it is possible that NKT cells play a distinct role in tumor immunity in different tissues. Moreover, type II NKT cells are most prevalent in the lungs and liver, two organs that are major sites of cancer metastases (28). Type II NKT cells play a critical role in immune suppression of tumor immunity against CT26 tumors in lungs, as NKT cell-deficient CD1d^−/−^ mice, but not type I NKT cell-deficient Jα18^−/−^ mice are resistant to tumor development (24, 107). Tregs do not play a major role in the immune regulation in this model, as anti-CD25 treatment does not alter tumor burden in the lungs (139). By contrast, Tregs rather than NKT cells play a critical role in suppression of tumor immunity against the same CT26 tumors growing in the skin. Therefore, against the same tumor, different T cells in different tissues regulate tumor immunity.

Recent studies suggest that induction of resident memory CD8^+^ T (T_RM_) cells is critical for the protection against tumors in mucosal tissues. Intranasal delivery of a cancer vaccine that induces T_RM_ is reported to reduce tumor progression of TC1 orthotopic head and neck or lung tumors in both prophylactic and therapeutic settings, while intramuscular systemic immunization does not (140, 141). The protection was not transferred from immunized mice to naïve mice through parabiosis, confirming the protective memory CD8^+^ T cells induced at the site of induction do not go into the circulation. Consistently, it has been reported that adoptive transfer of effector CD8^+^ T cells induced by s.c. injection of a DC vaccine loaded with tumor antigens could protect recipients against s.c. tumors but not against gastric tumors (142). In humans, recent studies report that the number of T_RM_ cells in tumors correlates with prolonged survival in a variety of types of cancers (143–149).

TGF-β plays a critical role in induction and retention of tissue-resident immune cells. Indeed, blockade of TGF-β, a cytokine required for the differentiation of T_RM_ cells, significantly reduced the number of T_RM_ induced by as well as protective effect of the mucosal vaccine against lung tumors (140). This is in contrast to the effect of anti-TGF-β treatment in some lung metastasis models in which the authors examined the effect of TGF-β blockade in tumor immunosurveillance (91, 100, 150, 151) and studies showing enhancement of tumor vaccine efficacy (98, 100, 152, 153). In addition, recent studies of cancer patients suggested that TGF-β is a factor negating the effect of PD-1 blocking checkpoint inhibitor treatment (101, 102). Further studies are required to provide potential explanations for the sometimes conflicting effects of TGF-β blockade in tumor immunity.

Since immunity against CT26 tumors in different tissues is regulated by different suppressive T cells, we asked whether the effector T cells against CT26 induced in lungs and skin by removing immune suppression are cross-protective against CT26 in the other tissue (139). Mice that rejected subcutaneous tumors after receiving anti-CD25 treatment rejected tumors not only in the skin on the contralateral side but also in the lungs, suggesting that the effector T cells induced in skin go into the systemic circulation. This was further confirmed by the observation that adoptive transfer of splenic T cells from the mice immune to subcutaneous tumors transferred the protection not only against subcutaneous tumors but also against lung tumors. By contrast, splenic effector T cells in CD1d^−/−^ mice immune to lung tumors could not transfer any protection against subcutaneous tumors, although they did against lung tumors. Thus, there is a unidirectional migration of protective memory T cells from skin to lungs (139). Protective memory T cells in this system are unlikely T_RM_, as the protection was transferred by splenic T cells. The observations in CT26 models were strikingly different from other studies suggesting that T_RM_ cell induction is crucial for the protection against mucosal tumors. The difference between this study with CT26 models and others is that all other studies induced memory T cells by using vaccines while this study with CT26 tumors in different tissues did not, but rather depended on natural immunosurveillance. As local APCs at the site of priming can determine tissue specificity of activated T cells, and Tregs can affect functions of APCs, it may be possible that APC’s ability to determine tissue specificity of induced T cells is changed in the absence of regulatory cells such as Tregs and type II NKT cells. The mechanism of this observation requires further elucidation.

## Conclusion

Here, we have seen that both type I and type II NKT cells play critical roles in tumor immunity. While most often type I NKT cells promote tumor immunity and type II NKT cells suppress it, type I NKT cells can also induce suppressive Tregs as well as protective NK cells. Moreover, both have a myriad of interactions with other immune effector and regulatory cells, forming a complex web of immune regulation. Type I and II NKT cells can cross-regulate each other, forming an immunoregulatory axis that comes into play early in immune responses. By regulating type II NKT cells, type I NKT cells also determine the balance between the latter and Tregs to determine which will dominate in controlling immunity within a particular tumor, regulating the regulators. This has clinical implications because when type I NKT cells are absent, both Tregs and type II NKT cells can suppress in the same tumor, and this situation mimics the situation often found in cancer patients, in which type I NKT cells are deficient in numbers or function. Thus, blockade of both simultaneously may be necessary. The finding that the immune regulation and antitumor effector mechanisms within the same tumor growing in different tissues, such as skin and lungs, are different is also clinically important. The immunotherapy required for a primary tumor may not be the best for metastases growing in a different tissue environment. Thus, understanding the complex multidimensional interactions among a large network of regulatory and effector cells, including type I and type II NKT cells, will be critical to design the most effective immunotherapies for cancer.

## Author Contributions

All authors listed have made a substantial, direct, and intellectual contribution to the work and approved it for publication.

## Conflict of Interest Statement

The authors declare that the research was conducted in the absence of any commercial or financial relationships that could be construed as a potential conflict of interest.
